# A polyaxial fixation brace for the treatment of idiopathic congenital talipes equinovarus in newborns

**DOI:** 10.1186/s13018-019-1268-9

**Published:** 2019-07-12

**Authors:** Yuxi Su, Yan Xie, Xiaopeng Kang, Guoxin Nan

**Affiliations:** 1 Department II of Orthopaedics, Chongqing Key Laboratory of Pediatrics, Ministry of Education Key Laboratory of Child Development and Disorders, China International Science and Technology Cooperation base of Child Development and Critical Disorders, Children’s Hospital of Chongqing Medical University, 136# Zhongshan 2 road, Chongqing, 400014 Yuzhong District China; 2 Clinical Laboratory Department, Maternal and Child Health Care Hospital of Chongqing Yubei District, No.71, Shuanghu branch road, Chongqing, Yubei District China; 3 Orthopaedics Department, Kunming Children’s Hospital, No. 288, Qianxing road, Xishan district, Kunming city, Yunnan Province China

**Keywords:** Congenital talipes equinovarus, Pirani score, Newborn, Polyaxial brace

## Abstract

**Background:**

Treatment of idiopathic congenital talipes equinovarus (CTEV) is challenging for pediatric orthopedic surgeons. The Ponseti method is an effective protocol for treatment due to its technique of manipulation, casting, and limited surgery. Plaster casting is an essential component of the Ponseti method. In this report, we describe a new brace that was developed for use in the treatment of clubfoot in newborns instead of a plaster cast.

**Methods:**

This retrospective study was performed in two orthopedic medical centers. Between January 2011 and October 2013, 89 newborns with CTEV (131 ft) underwent corrective treatment using fixation braces in the experiment group (E-group) in our hospital, and 107 newborns with CTEV (141 ft) underwent plaster casting in the control group (C-group) in another medical center. All patients were treated according to the Ponseti method after the application of the inclusion and exclusion criteria. Plaster casts were applied to patients in the C-group. The patients in the E-group received the custom-made polyaxial fixation braces instead of plaster casts. Prospective follow-up was performed for a mean duration of 36 months. The efficacy of the treatment was assessed using Pirani’s scoring system. Chi-squared and independent *t* tests were used for statistical analyses.

**Results:**

In the E-group, 85 patients (125 ft) achieved good appearance within 3 months of treatment initiation (average, 1.7 months). Four patients (6 ft) required percutaneous Achilles tenotomy. Seven patients developed sores during treatment because of improper brace application, but all sores healed without scarring with timely treatment. In the C-group, 96 patients (123 ft) achieved good appearance within 3 months of treatment initiation (average, 1.6 months). Eleven patients (18 ft) required percutaneous Achilles tenotomy. Twenty-one feet developed sores during treatment because of plaster cast pressure on the dorsum of the feet. Sixteen sores healed without scarring with timely treatment, and 5 ft had obvious scars. The overall mean Pirani scores 1 year after treatment were 0.26 ± 0.06 in the E-group and 0.25 ± 0.03 in the C-group, and the Pirani scores 3 years after treatment were 0.23 ± 0.05 in the E-group and 0.22 ± 0.03 in the C-group. There were significant differences in the percutaneous Achilles tenotomy and skin sores but no significant difference in the Pirani scores between these two groups.

**Conclusions:**

Our results showed that the new polyaxial fixation brace used in this study was an effective tool for the corrective treatment of CTEV in newborns. We propose the use of this brace as an alternative treatment for newborns.

## Introduction

CTEV, also known as clubfoot, is the fifth most common congenital malformation in children [1]. CTEV consists of four components: equinus, heel varus, forefoot adduction, and cavus [2]. CTEV occurs as an isolated birth defect with no other malformations. The etiology of CTEV is largely unknown. If CTEV is associated with secondary or syndromic or another congenital disease, it is defined as secondary CTEV. These patients account for 20% of all CTEV patients [3]. In our study, we focused on patients without secondary CTEV because secondary CTEV seems to derive from neuromuscular or fetal abnormalities involved in its etiopathogenesis, which makes it rather different in clinical presentation, treatment, and proposed etiopathogenetic mechanism [3]. There are different methods available for the treatment of clubfoot. The treatment with the best long-term success rate is the Ponseti method [4]. The Ponseti technique has been used for more than 50 years, and it is accepted worldwide because extensive open surgery is commonly associated with long-term stiffness and weakness, which are avoided with the use of the Ponseti technique [4, 5]. Most orthopedic surgeons agree that the initial treatment for CTEV should be nonsurgical and should be initiated as soon as possible after birth [6, 7]. The Ponseti method of CTEV management has been shown to be effective, producing better results and fewer complications than traditional surgical approaches [8, 9]. A 10-year follow-up study also proved that the Ponseti method resulted in better ankle power and isokinetic strength for clubfeet compared with open surgery methods. This method consists of a weekly program of manual manipulation and above-knee plaster casting. Plaster casting is an essential part of this treatment [10]. We recently developed a simple brace for use in newborns and infants that produced excellent results [11]. We developed a second-generation brace with three polyaxial joints that can be easily adjusted for proper foot positioning after manipulation. The present study used this new fixation brace in the treatment of CTEV in newborns and assessed its efficacy.

## Materials and methods

### Patients

This study included 89 consecutive patients (55 males, 34 females, 131 clubfoot deformities, bilateral in 42 patients) in the E-group who underwent the previously described manipulation and brace fixation treatment protocol, and 96 patients (57 males, 39 females, 123 clubfeet deformities, bilateral in 27 patients) in the C-group who underwent the traditional Ponseti method, between January 2011 and October 2013. The inclusion criteria were patients who initiated their treatment less 28 days after birth and were diagnosed with unilateral or bilateral idiopathic CTEV deformity. The exclusion criteria were newborns who were treated more than 28 days after birth, newborns with acquired CTEV deformity secondary to other disorders or syndromes, newborns with developmental delays associated with neuromuscular disorders, or newborns treated by another medical institute. The patients were first seen by orthopedic surgeons and recruited into the study. The ethics committee of our hospital approved the study. The parents or guardians of the patients signed an informed consent form before participation in the study and authorized the publication of the study results and the use of photographs of their children. Trial registration: NCT02815306. Retrospectively registered, https://www.clinicaltrials.gov/ct2/show/NCT02815306?cond=NCT02815306&rank=1

### Treatment process

The detailed technique was in full accordance with the Ponseti method [6]. The treatment phase began at the time of diagnosis. The complete treatment process was described in detail in a printed manual given to the patients’ caregivers. A trained, certified therapist initially performed the manual manipulations and instructed the caregivers until they were able to manipulate the foot and apply the brace properly. Caregivers were told to check the skin every 3 to 5 h for the first week to prevent pressure sores. Because the first month is the most important period in the treatment process, patients were seen in the outpatient clinic at least once a week during this time. At these visits, the orthopedic surgeon evaluated the patients and reinstructed caregivers if the results were not as desirable as expected. Patients were seen regularly at the outpatient clinic once a month for follow-up after the first month of treatment, initially to monitor for brace complications and then to identify possible residual deformities. The indication for tenotomy was the same as the Ponseti method: correct equinus when cavus, adductus, and varus were fully corrected but ankle dorsiflexion remained less than 10° above neutral. Patients who were treated for over 3 months, i.e., patients who exhibited tenotomy indication, were treated with percutaneous Achilles tendon tenotomy. If the correction was still not sufficient, then the following surgeries were performed: Achilles tendon lengthening, posterior capsulotomy, tibialis posterior lengthening, capsulotomy of the talo-navicular joint, or lengthening of flexor hallucis longus or flexor digitorum tendon [12]. The Denis Browne splint was used with the Ponseti method after Achilles tendon tenotomy.

The procedure used for manual correction was the same as the Ponseti method. One caregiver held the patient’s calf, while another caregiver held the patient’s forefoot with their right hand to stabilize the talus by placing their left thumb over the lateral part of the talus head and elevating the first ray to achieve supination of the forefoot with respect to the mid- and hind-foot. This procedure was maintained for 5–10 min and was repeated with intervening short breaks. The brace was applied full time, except during manipulation sessions. The brace was adjusted according to the patient’s food deformity until forefoot adduction was corrected to a neutral or overcorrected position.

Next, during the maintenance phase (duration, 1–3 years), a Denis Browne splint was used at night to prevent recurrence [13]. Participants were fully informed of the treatment plan prior to the start of treatment and confirmed that the plan would be followed.

### Polyaxial fixation brace construction

**Fig. 1:**
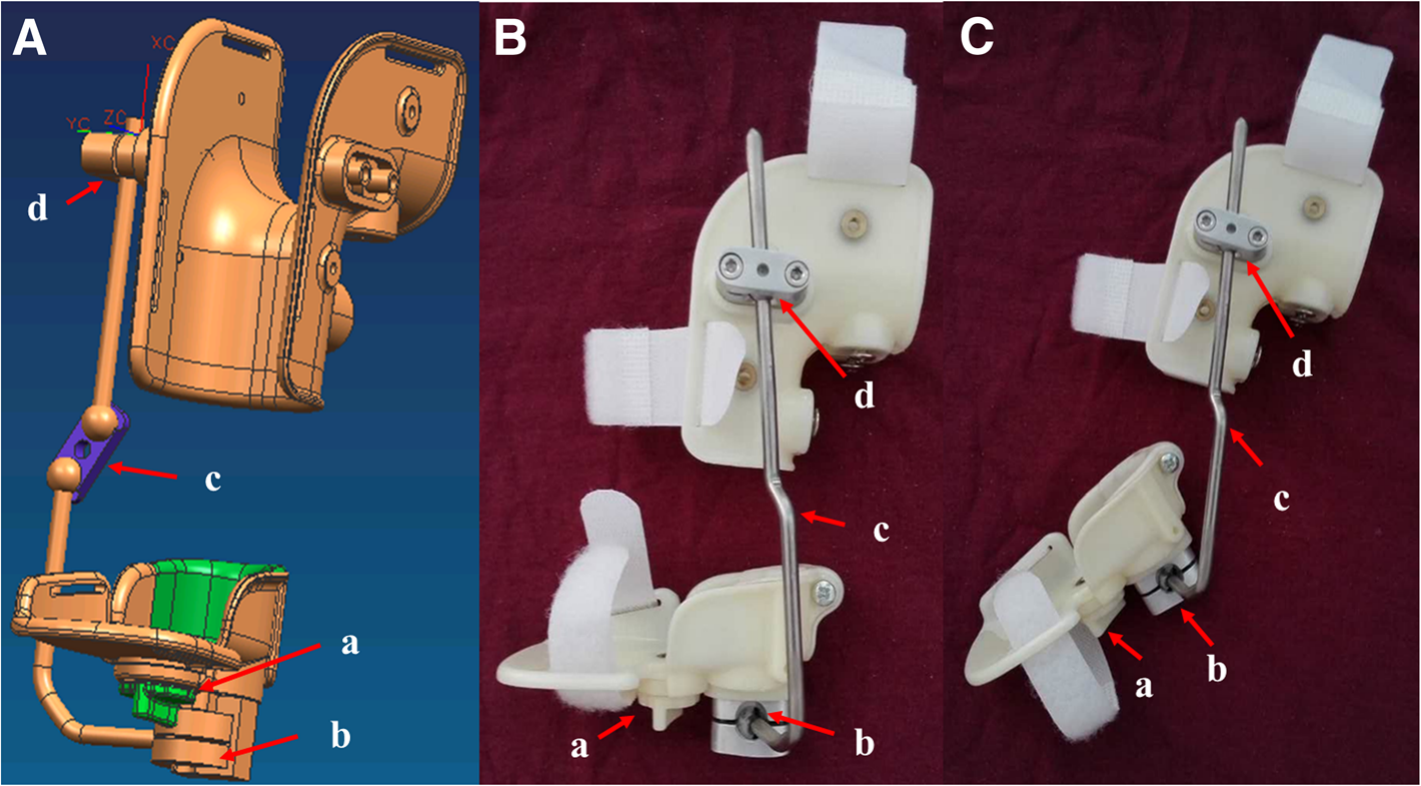
The principle of the polyaxial braces. **a** The design of the brace; there are three critical joints that help fix the foot at all angles. **b**, **c** The actual brace made by a professional technician

**Fig. 2:**
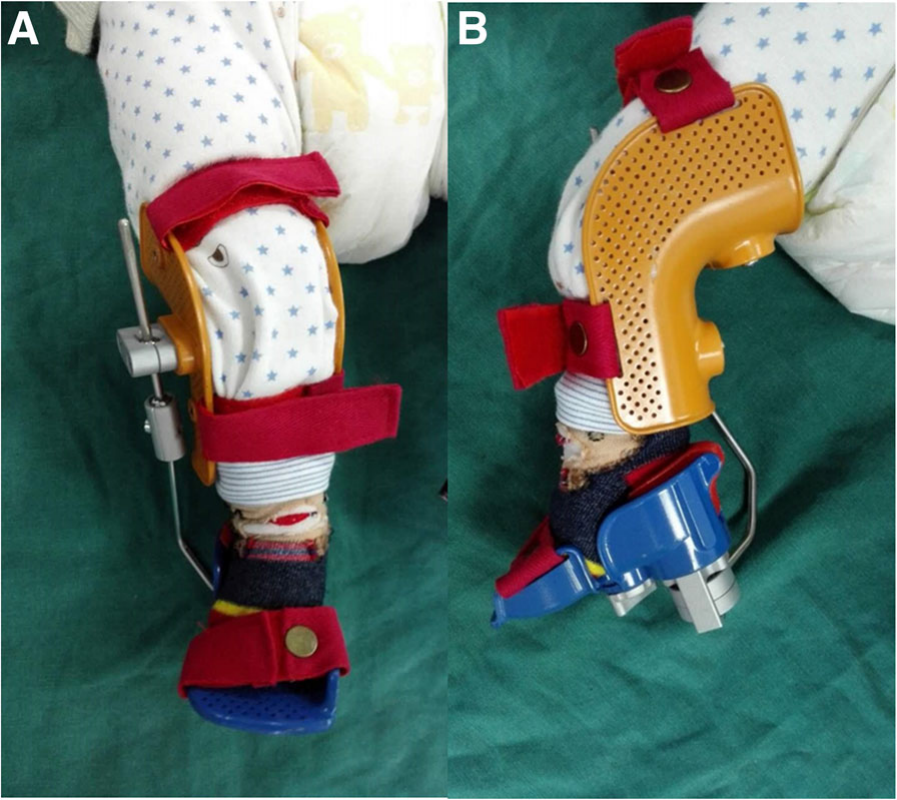
A 2-month-old patient with a polyaxial fixation brace on the right foot. **a**, **b** The frontal and lateral sides, respectively

The braces were made by a technician trained by the National Disabled Persons Federation. A Gypsum model was prepared first, and braces were fabricated according to the model for each patient. The brace possessed three different joints: (1) Joint a (located at the bottom of the brace, *a* in Fig. 1a) was used primarily for correction of the forefoot adductus; (2) Joint b (located lateral to the ankle, *b* in Fig. 1a) was primarily involved in correction of the hindfoot varus deformity; and (3) Joints c and d (located at the top of the brace, c, d in Fig. 1a) were primarily responsible for the correction of the ankle equinus deformity. All of the joints were adjustable according to the appropriate degree of correction needed. With the rapid growth of the infants, braces were replaced when they no longer fit properly (Fig. 2).

### Measures

All patients in both groups were assessed consecutively and prospectively using the same standard, when they were newborns (first diagnosed, preoperatively), at 1 month, 3 months, and 1 year postoperatively, and at the age of 3 years (final evaluation). All patients were assessed using Pirani’s scoring system before treatment and during outpatient follow-up visits. This system has six variables (posterior crease, empty heel, equinus, and reduction of the navicular bone, medial crease, and lateral curvature of the foot), and each variable is scored as 0, 0.5, or 1, with 1 indicating maximum deformity. A foot with maximum deformity has a total score of 6, and a normal foot has a total score of 0.

### Statistical analysis

Each foot was used as an independent observation in statistical analyses. Statistical analysis was performed using the SPSS 22.0 software package (SPSS, Chicago, IL, USA), and the data are given as the means ± standard deviation (SD). The chi-squared and independent *t* tests were used in the statistical analyses, and a value of *p* < 0.05 was considered statistically significant.

## Results

### Patient information

The average ages of treatment initiation were 5.3 days after birth (range, 0 to 28 days) in the E-group and 5.7 days (range, 0 to 28 days) for the C-group (*P* = 0.8878). The mean initial Pirani scores before treatment were 4.60 ± 1.04 (3.0–6) points for the E-group and 4.66 ± 0.88 for the C-group, and there was no significant difference between these groups (Table 1). Twenty-one patients (11 in the E-group, 10 in the C-group) were lost to follow-up during the study. The final analyses were based on complete follow-up data for all patients who were followed up eventually.

**Table 1:** Patients’ age, gender, Pirani scores, and complications

	E-group	C-group	*P* value #
Age			
Mean age (days)	7.5 ± 5.6	8.1 ± 4.9	0.87
Gender			
Male	55	57	
Female	34	39	
Quantity of feet	131	123	
Bilateral	42	27	
Pirani scores*			
Postoperative	4.60 ± 1.04	4.66 ± 0.88	0.55
1st month	2.35 ± 1.88	1.24 ± 1.69	0.021
3rd month	1.33 ± 0.75	0.45 ± 0.35	0.012
6th month	0.44 ± 0.55	0.31 ± 0.32	0.27
12th month	0.26 ± 0.06	0.25 ± 0.03	0.48
36th month	0.23 ± 0.05	0.22 ± 0.03	0.57
Complications (36 ± 1.3 months)			
Skin pressure sores	7	21	0.0283
Scars	0	5	0.0283
Percutaneous Achilles tendon lengthening	4	18	0.0109
Slippage	0	21	0.0001
Posterior capsulotomy	1	8	0.0001

*According to the Pirani score evaluation standard

^#^*p* < 0.05 was considered statistically significant

### Treatment outcomes

**Fig. 3:**
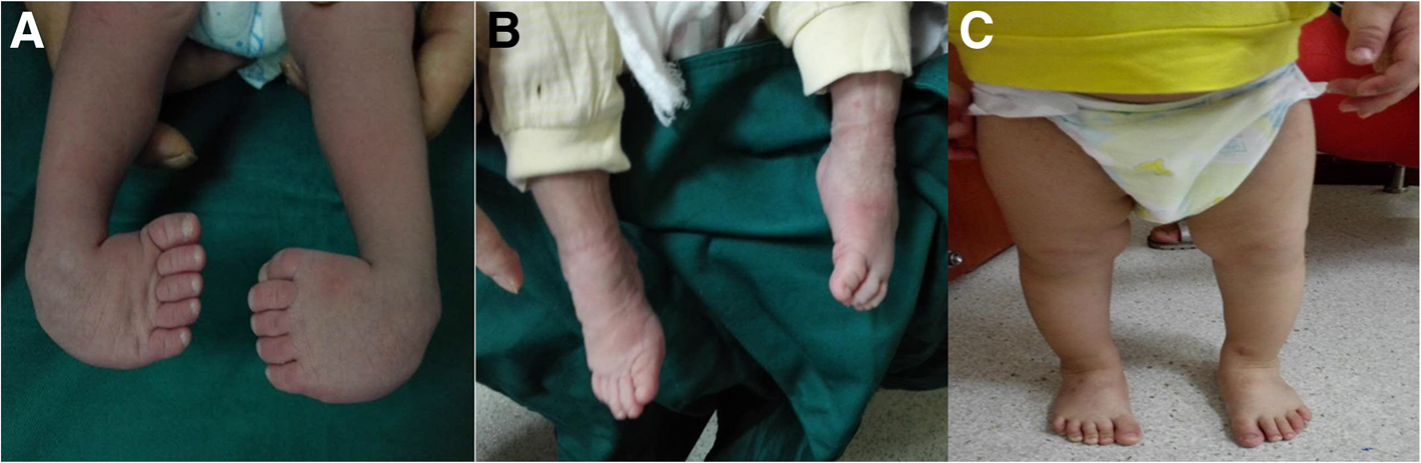
**a** A newborn infant who was diagnosed with congenital clubfoot and started treatment at 14 days of age. **b** The degree of forefoot pronation achieved after 1 month. **c** The normal appearance of the foot 3 months later

All patients were followed for 35–37 months, with an average follow-up period of 36 months. The deformities were corrected to normal after an average treatment duration of 2.6 months (range, 1.5–3 months), after which time patients began using a Denis Browne splint. Skin pressure sores were seen in 7 patients in the E-group due to improper brace care, but no scarring occurred following timely treatment. Four (6 ft) of these patients were treated with percutaneous Achilles tendon lengthening. The overall mean Pirani scores 1 year after treatment were 0.26 ± 0.06 and 0.23 ± 0.05 in the third year follow-up, which were significantly lower than before the treatment (*P* = 0.0052; Table 1). Twenty-one feet developed sores during treatment in the C-group because of plaster cast pressure on the dorsum of feet. Sixteen feet healed without scarring with timely treatment, and 5 ft developed obvious scars, which delayed the treatment of their CTEV for almost 2 months. Eleven patients (18 ft) required percutaneous Achilles tenotomy. The overall mean Pirani scores 1 year after treatment were 0.25 ± 0.03 and 0.22 ± 0.03 in the third year follow-up. There were no significant differences between the E-group and the C-group in the Pirani scores, but there was a significant difference in complications. Photographs of representative patients are shown in Fig. 3.

## Discussion

Although controversy remains regarding the management of clubfoot, the Ponseti treatment method has been shown in recent years to be more effective than the traditionally used surgical methods. Recent studies suggest that the Ponseti method is successful in up to 98% of cases. The use of the Ponseti method allows patients to avoid the complications of surgery and leads to the normal appearance and function of the congenital clubfoot. The deformity is reduced by weekly manipulation and plaster casting, which is an essential component. Aydin et al. [10] found that slippage and skin lesions were significantly more common with plaster of Paris casts compared to a semirigid synthetic soft cast and were associated with lower parent satisfaction. We report the development of a new brace designed with three polyaxial joints, as shown in Fig. 1. We believe this brace can be used in place of plaster and semirigid synthetic soft casts. We applied the brace to newborns who were diagnosed with CTEV.

The age at which to start treatment is controversial, with some authors arguing that casting according to the Ponseti method should begin in infants older than 1 month of age or with an involved foot ≥ 8 cm in length [10, 14]. Zionts et al. [10] emphasized that treatment of CTEV should not be considered an orthopedic emergency and contended that newborns and infants have more complications, such as cast slippage, cast- and brace-related skin problems, early noncompliance with brace wearing, and relapse before 1 year. Our experience suggests that the newly designed braces solve these problems. Our experience also suggests that the earlier we started the treatment, the better results we could obtain [11].

The patients included in this study were all newborns less than 28 days old. There were significant differences in the 1st, 2nd, and 3rd months between the C-group and the E-group according to the Pirani scoring evaluations. The Pirani scoring system gave no functional measurement, and it was purely descriptive of the deformity. There was a significant difference between these two groups in Pirani scores at 6 months. However, complications, such as skin pressure sores, percutaneous scars, Achilles tendon lengthening, and slippage, exhibited significant differences between these two groups. This result may be due to the more moderate manipulation and fewer complications in the E-group than the Ponseti method.

There are some disadvantages to the traditional Ponseti method. First, a manual correction occurs weekly with plaster changes instead of daily [6]. Second, once the plaster is applied, it is difficult to determine the condition of the skin. This factor is especially significant for newborns with delicate skin and the inability to communicate their discomfort. When pressure ulcers are recognized, therapy is interrupted, and parental satisfaction is decreased. Third, the plaster easily slips off the lower limb if the physician applying it is not adequately trained and experienced. Fourth, bathing is not allowed during the treatment. Fifth, plaster fixation can be very uncomfortable in a hot and humid climate. However, plaster casting is simple and inexpensive for use in developing countries with limited financial and social resources for health services [15].

When using the new polyaxial fixation brace, just as with the Ponseti method, therapists and caregivers must strictly adhere to a gradual, ordered treatment plan. Otherwise, the outcome may be a flatfoot or rocker bottom foot deformity. Continuous force causes the ligament and joint capsules to gradually relax, and the brace may be adjusted at the three joints and locked into place. We found that the patients experienced almost no pain from the brace and that any skin lesions that developed were easily observed and treated. The fixation brace can be removed at any time, which makes skin care much more convenient.

Manual correction is mainly focused on correcting the forefoot adduction, varus deformity, and hindfoot cavus, in that order, followed by correction of the equinus. All braces used in this study were custom-made. When we made the first generation brace, the correction degree of the brace varied in accordance with the deformity of the foot. For the new generation model, the brace needs to fit a patient’s foot, ankle, and leg. However, there is no need to consider the deformity angles because the three joints are adjustable. For the safety and protection of the heel of the foot, a silica gel was added to the hindfoot area of the brace.

Tenotomy may be needed with the Ponseti treatment method [16], and this need can sometimes be predicted [17]. Only 4 patients and 6 ft tenotomy procedures were required in our study, and they are all the resistant CTEV [18], which needed the surgery for correction [16].

It is plausible that the brace could be used at night until preschool, which occurs at approximately 7 years old [8]. A foot abduction brace is a crucial part of the Ponseti treatment, and it is considered mandatory to prevent relapse [19, 20]. There were 3 patients (5 ft) in the E-group and 11 patients (14 ft) in the C-group who experienced relapse 3–5 months later. These patients had to restart the procedure from the very beginning using the same methods.

The key to the efficacy of this treatment method is having the cooperation of the caregivers [21]. Adherence to brace use is the most important part of the Ponseti method [21], and adherence is correlated with the caregivers’ education [22]. We found that urban parents had better adherence than rural parents. Although doctors can provide individualized treatment plans for patients, caregivers are the executors of these plans during the treatment process. Therefore, caregivers should be adequately trained to perform manipulations. The therapeutic schedule should be carefully explained to produce confidence in caregivers and increase their cooperation with the treatment. Caregivers must be informed to watch children closely and to remove the brace to check for possible compression injury of the skin during the initial period of brace fixation.

Our experience suggests that the use of the polyaxial fixation brace is safe, comfortable, and conducive to observation and skin care. The polyaxial fixation brace provides another choice for the treatment of CTEV. However, the present study enrolled patients younger than 1 month, and research is needed on older infants and children. A prospective, randomized, control clinical trial may be needed for further research.

## Conclusion

The results of this study indicate that the new polyaxial brace can be used instead of casting for the effective treatment of CTEV in newborns. However, a longer follow-up period is needed to evaluate the long-term efficacy of the polyaxial fixation brace in clubfoot treatment.

## Data Availability

Please contact the authors for data requests.

## Abbreviations

C-group: Control group
CTEV: Congenital talipes equinovarus
E-group: Experiment group

## References

[1] Ostadal M, Chomiak J, Dungl P, Frydrychova M, Burian M (2013). Comparison of the short-term and long-term results of the Ponseti method in the treatment of idiopathic pes equinovarus. Int Orthop.

[2] Ponseti IV (1994). The treatment of congenital clubfoot. J Orthop Sports Phys Ther.

[3] Pavone V, Chisari E, Vescio A, Lucenti L, Sessa G, Testa G (2018). The etiology of idiopathic congenital talipes equinovarus: a systematic review. J Orthop Surg Res.

[4] Pavone V, Testa G, Costarella L, Pavone P, Sessa G (2013). Congenital idiopathic talipes equinovarus: an evaluation in infants treated by the Ponseti method. Eur Rev Med Pharmacol Sci.

[5] Jeans KA, Karol LA, Erdman AL, Stevens WR (2018). Functional outcomes following treatment for clubfoot: ten-year follow-up. J Bone Joint Surg Am.

[6] Ponseti IV (2002). The ponseti technique for correction of congenital clubfoot. J Bone Joint Surg Am.

[7] Changulani M, Garg NK, Rajagopal TS, Bass A, Nayagam SN, Sampath J (2006). Treatment of idiopathic club foot using the Ponseti method. Initial experience. J Bone Joint Surg Br.

[8] Scher DM (2006). The Ponseti method for treatment of congenital club foot. Curr Opin Pediatr.

[9] Derzsi Z, Nagy O, Gozar H, Gurzu S, Pop TS (2015). Kite versus Ponseti method in the treatment of 235 feet with idiopathic clubfoot: results of a single Romanian medical center. Medicine (Baltimore).

[10] Aydin BK, Sofu H, Senaran H, Erkocak OF, Acar MA, Kirac Y (2015). Treatment of clubfoot with Ponseti method using semirigid synthetic softcast. Medicine (Baltimore).

[11] Su Y, Nan G (2014). Manipulation and brace fixing for the treatment of congenital clubfoot in newborns and infants. BMC Musculoskelet Disord.

[12] Grigoriou E, Abol Oyoun N, Kushare I, Baldwin KD, Horn BD, Davidson RS (2015). Comparative results of percutaneous Achilles tenotomy to combined open Achilles tenotomy with posterior capsulotomy in the correction of equinus deformity in congenital talipes equinovarus. Int Orthop.

[13] Chen W, Pu F, Yang Y, Yao J, Wang L, Liu H (2015). Correcting congenital talipes equinovarus in children using three different corrective methods: a consort study. Medicine (Baltimore).

[14] Iltar S, Uysal M, Alemdaroglu KB, Aydogan NH, Kara T, Atlihan D (2010). Treatment of clubfoot with the Ponseti method: should we begin casting in the newborn period or later. J Foot Ankle Surg.

[15] Morcuende JA, Cook TM (2015). The Ponseti method in low and middle income countries: challenges and lessons learned. Foot Ankle Clin.

[16] Kowalczyk B, Felus J (2015). Ponseti casting and Achilles release versus classic casting and soft tissue releases for the initial treatment of arthrogrypotic clubfeet. Foot Ankle Int.

[17] Aydin BK, Senaran H, Yilmaz G, Acar MA, Kirac Y (2015). The need for Achilles tenotomy in the Ponseti method: is it predictable at the initiation or during the treatment. J Pediatr Orthop B..

[18] Ponseti IV, Zhivkov M, Davis N, Sinclair M, Dobbs MB, Morcuende JA (2006). Treatment of the complex idiopathic clubfoot. Clin Orthop Relat Res.

[19] Ramírez N, Flynn JM, Fernández S, Seda W, Macchiavelli RE (2011). Orthosis noncompliance after the Ponseti method for the treatment of idiopathic clubfeet: a relevant problem that needs reevaluation. J Pediatr Orthop.

[20] Solanki PV, Sheth BA, Poduval M, Sams SB (2010). Effectiveness of modified ankle foot orthosis of low-temperature thermoplastics in idiopathic congenital talipes equino varus. J Pediatr Orthop B.

[21] Goksan SB, Bilgili F, Eren I, Bursali A, Koc E (2015). Factors affecting adherence with foot abduction orthosis following Ponseti method. Acta Orthop Traumatol Turc.

[22] Avilucea FR, Szalay EA, Bosch PP, Sweet KR, Schwend RM (2009). Effect of cultural factors on outcome of Ponseti treatment of clubfeet in rural America. J Bone Joint Surg Am.

